# Detecting Near-Surface Sub-Millimeter Voids in Additively Manufactured Ti-5V-5Al-5Mo-3Cr Alloy Using a Transmit-Receive Eddy Current Probe

**DOI:** 10.3390/s24134183

**Published:** 2024-06-27

**Authors:** Brendan Sungjin Halliday, Allyson Eastmure, Peter Ross Underhill, Thomas Walter Krause

**Affiliations:** 1 Department of Physics, Engineering Physics and Astronomy, Queen’s University, Kingston, ON K7L 3N6, Canada; 17bsh3@queensu.ca (B.S.H.); 17aie@queensu.ca (A.E.); 2 Department of Physics and Space Science, Royal Military College of Canada, Kingston, ON K7K 7B4, Canada; ross.underhill@rmc.ca

**Keywords:** Ti-5V-5Al-5Mo-3Cr, additive manufacturing, transmit-receive eddy current, subsurface voids

## Abstract

Additive Manufacturing (AM) Direct Laser Fabrication (DLF) of Ti-5Al-5V-5Mo-3Cr (Ti5553) is being developed as a method for producing aircraft components. The additive manufacturing process can produce flaws near the surface, such as porosity and material voids, which act as stress raisers, leading to potential component failure. Eddy current testing was investigated to detect flaws on or near the surface of DLF Ti5553 bar samples. For this application, the objective was to develop an eddy current probe capable of detecting flaws 500 µm in diameter, located 1 mm below the component’s surface. Two initial sets of coil parameters were chosen: The first, based on successful experiments that demonstrated detection of a near surface flaw in Ti5553 using a transmit-receive array probe, and the second, derived from simulation by Finite Element Method (FEM). An optimized transmit receive coil design, based on the FEM simulations, was constructed. The probe was evaluated on Ti5553 samples containing sub-surface voids of the target size, as well as samples with side-drilled holes and samples with holes drilled from the opposing inspection surface. The probe was able to effectively detect 80% of the sub-surface voids. Limitations included the probe’s inability to detect sub-surface voids near sample edges and a sensitivity to surface roughness, which produces local changes in lift-off. Multifrequency mixing improved signal-to-noise ratio when surface roughness was present on average by 22%. A probe based on that described in this paper could benefit quality assurance of additively manufactured aircraft components.

## 1. Introduction

### 1.1. Background and Motivation

Additive Manufacturing (AM) Direct Laser Fabrication (DLF) Technology is being examined for the efficient manufacturing of complex Ti-5Al-5V-5Mo-3Cr (Ti5553) aircraft components. DLF creates components layer by layer by heating powder grains, delivered by a coaxial nozzle, to a molten state and depositing it on a previously solidified layer [1]. This process allows for faster manufacturing, the ability to manufacture without tools, manufacture of complex geometries and creates a metastable composition [1,2]. More broadly, the advantages of AM could also positively impact supply chain efficiency as single body complex geometries produced by AM require fewer individual parts [3,4]. However, the process of additive manufacturing may result in near subsurface flaws, such as voids and porosity that are difficult to detect using conventional inspection methods. Porosity is generally accepted as being caused by lack of fusion during the laser fabrication process [5]. These flaws can act as a stress raiser and lead to decreased fatigue life and thereby, lead to component failure [6]. Other morphological defects that may occur during the DLF AM process are increased surface roughness and surface warpage [7,8]. These types of defects make inspecting previously mentioned internal defects (voids and porosity) difficult to detect, especially in the case of Ultrasonic inspection (UT) [9].

UT is widely considered one of the most versatile forms of non-destructive testing as it is not limited by material conductivity. The challenge with UT, however, is its limited ability in detecting near surface flaws due to front wall echoes. UT can detect near surface flaws if an appropriate back wall reflection is available. However, when complex geometries are made using AM, back wall reflections may not be accessible. It has been shown that UT can detect near surface flaws despite front wall echoes by using post-processing with data analysis techniques [10]. While possible, this process is not done in real time and is not suited for fast initial inspections [10]. Other forms of UT inspection of AM materials include Laser Ultrasonics (LU) [11]. LU is a form of non-contact UT inspection that uses laser pulses to induce acoustic waves in a material through the rapid thermal expansion of surface material. Xu et al. [11] used LU for detecting sub-millimeter drill holes in AM Al-Si-10Mg alloy, 316L alloy, Ti-6Al-4V alloy and In718 alloy at depths of 3 mm from the inspection surface. However, the surface roughness encountered in Xu et al. [11] was at most 0.6 µm, whereas the maximum roughness variance encountered in this paper was measured to be 80 µm. This level of surface roughness may cause issues for UT as increased surface roughness results in increased front wall scattering and thus decreased signal amplitude [12]. Additionally, through destructive testing seen later in this study, it was confirmed that the subsurface voids are of an irregular geometry. Irregular geometric defects are challenging to detect with UT, since the randomly oriented edges of the defect scatter acoustic waves in unpredictable directions [13].

To address these inspection needs, eddy current testing was examined for its potential in detecting these types of flaws. Eddy current testing (ECT) is a non-destructive testing (NDT) method that can be used to detect surface-breaking and near-surface flaws within a conductive material [14]. This allows manufactured components to be individually tested before they are put into service, without causing any damage to the component itself. Little work has been done on detecting and characterizing near-surface voids on sub-millimeter scale using ECT. Cecco et al. [14] describes some ways in which sub-surface flaws can be detected, although this literature does not thoroughly address near-surface voids for ECT in a transmit-receive configuration. 

Other forms of ECT, such as Pulsed Eddy Current (PEC) inspection, have been successful in detecting sub-surface flaws in a variety of settings. In Babbar et al. [15], PEC techniques, paired with modified Principal Component Analysis (PCA) have been successful in detecting far side OD defects in ferromagnetic Steam Generator (SG) tubing. Tube fretting was as close as 0.03 mm from the inner tube wall surface in this application. However, far-side flaws are an inherently different type of flaw compared to a sub-surface void. A definition for a sub-surface void would be a material discontinuity that allows eddy current to pass over, under and around it. Far-side flaws only allow eddy current to pass above them (relative to the coil surface), and thus induce a different EC response from that of a sub-surface void. Stott et al. [16] demonstrated a more comparable situation, demonstrating detection of Electrical Discharge Machining (EDM) notches emanating transversely around ferrous fasteners in the second layer of aluminum aircraft wing lap joints. These subsurface defects were anywhere from 0.9 to 5.5 mm in length and did not break the far surface. Stott et al. [16] used Principal Component Analysis (PCA) on the initial PEC response. While these techniques proved effective in detecting subsurface flaws in higher conductivity layered media [16], the much lower conductivity of Ti5553 (181 ± 1 µΩ·cm as measured here) will substantially shorten response time making it too short to discriminate near subsurface flaws [17]. 

In terms of eddy current technology, eddy current arrays in transmit-receive (T/R) configuration have been used to detect far side flaws in SG tubing. Specifically in Sullivan [18], an Eddy Current Testing Array (ETA) probe in T/R configuration, known as the X-probe, was modelled and built for detecting far-side circumferential flaws in 13 mm diameter Inconel 600 SG tubing. Sullivan’s [18] results demonstrated accurate detection and characterization of these flaws in this setting. The T/R configuration was used for its increased signal-to-noise ratio (SNR) for detecting far-surface flaws and increased differentiability of lift-off from defect signal [19,20]. 

Instances where ECT have been used to examine AM metal components are limited. Gel’atko et al. [21] inspected AM Stainless Steel (SS) 316L using absolute single coil ECT probes. Some of the artificial defects inspected were bottom drill holes and spherical cavities. Gel’atko et al. [21] showed detection of spherical cavity defects 0.5 mm in diameter as far as 1.5 mm from the surface. However, Gel’atko et al.’s [21] results show relatively low SNR for these types of flaws. The resistivity of SS 316L is 74 µΩ·cm [21,22], significantly less than the resistivity of Ti5553. This means depth of penetration is greater in Ti5553; however, eddy current density is reduced resulting in decreased sensitivity to similar flaws [17]. This suggests an absolute single coil ECT probe may not be suitable for inspecting Ti5553 due to its low SNR compared to other ECT probe configurations. 

ECT inspection applied to AM inspection was done by Spurek et al. [23], where a conventional impedance eddy current probe was integrated with a Powder Bed Fusion machine for in-situ measurements of localized electrical conductivity in Al-Si-10Mg. Conductivity measurements were used as a proxy for density; thus, manufacturing could be stopped mid-process if density decreased due to increased porosity. Though shown to be effective, this process was not designed for flaw detection. In this study, AM parts provided by the manufacturer were examined for post-processing inspection. The target flaw sizes were 100 µm and 500 µm. However, only samples containing 500 µm diameter voids were investigated. The introduction of such flaws does not represent porosity, especially when considering more modern AM methods [24]. However, the potential introduction of such a flaw would severely compromise a component’s integrity. Due to the relative novelty of AM, little work has been done on understanding how ECT will respond to subsurface voids in AM material, with the application of ECT in a transmit-receive configuration being largely absent. 

In this paper, it is demonstrated that a novel design based on Finite Element Method (FEM) calculations can detect near-surface, sub-millimeter voids in additively manufactured Ti5553. This eddy current probe uses a transmit-receive configuration due to its predicted increase in signal-to-noise ratio [19,20] particularly in the presence of lift-off [25]. Obtaining a high SNR is paramount as it is expected that a surface roughness variance of 80 µm acts as shallow surface breaking flaws, resulting in small vertical signal responses as measured with respect to lift-off. The defining limitation of this transmit-receive EC probe was difficulty in detecting subsurface flaws in the presence of high surface roughness. AM subsurface voids have potential applications in generating reproducible calibration defects for NDT inspections. For instance, a challenge in NDT inspection is generating calibration defects that are representative of inclusions. The probe developed and studied in this paper could also be used for inspecting such defects. 

### 1.2. Theory

Eddy current testing is a method that uses a probe in which alternating current flows through a wire coil, generating a time-dependent magnetic field. When the probe and its magnetic field are brought close to a conductive material, eddy currents or the circular flow of electrons through a material are generated according to Faraday’s Law [26,27,28]. If the circulation of the eddy currents is disturbed by a material defect or discontinuity, a signal can be observed via a change in the coil’s measured impedance. All equations considered in this report assume a coil is above a conducting infinite half space. 

In a conductor, Ohm’s Law states that the current flux density J is equal to the electric field multiplied by the conductivity σ, namely [27]:

(1) J=σE.

In the case where E is a time varying alternating electric field with angular frequency ω, and σ≫ωϵ is sufficiently small (less than GHz range [17]), we are left with the diffusion equation:

(2) $$\nabla^{2}J=\mu \sigma \frac{\partial J}{\partial t}.$$

For continuously time-varying conditions, current density then becomes [17]:

(3) $$J=J_{s}e^{-\frac{z}{\delta }}e^{i(\frac{z}{\delta }-\omega t)},$$

where z is the depth below the surface of the conducting half space, $J_{s}$ is the current flux density at the surface of the conductor and δ is the skin depth. The skin depth δ can be expressed as [17]:

(4) $$\delta =\sqrt{\frac{2}{\mu \sigma \omega }},$$

where μ is the material permeability. Skin depth is the depth of penetration at which J decreases by a factor of 1/e of its surface value. 

In practice, detecting defect signals on an impedance display requires that the defect signal be separated from other measured signals such as lift-off or environmental changes such as presence of support structure. Lift-off (separation between coil and conducting surface) can vary during a scan, resulting in a change in impedance of the pick-up coil. This signal is reproducible with a well-defined trajectory on the impedance plane. Lift-off signal can be rotated by an arbitrary phase angle such that the trajectory is aligned in the negative horizontal direction on the measurement display. Once rotated, any sufficiently large signal that is measured in the vertical component can be interpreted as not being affected by lift-off (perhaps a localized defect signal). An empirically derived result defines a frequency, $f_{90}$, that separates the lift-off signal and change-in-wall-thickness signals by a 90° phase angle [14]. The equation for $f_{90}$, for a single surface riding coil, is expressed in kHz as [14]:

(5) $$f_{90}=1.6\frac{\rho }{t^{2}},\;$$

where ρ is the material resistivity in µΩ·cm and t is the thickness in mm. It is claimed that thickness t can stand for defect depth z of a subsurface void [14]. The $f_{90}$ frequency is not the only consideration when choosing the optimal inspection frequency for flaw detection. The skin depth, as discussed in the previous section, plays an important role in flaw detection as well. A small δ implies eddy currents will decay quickly with depth. If the flaw is deeper than the skin depth, eddy current flux density will only interact weakly with the discontinuity resulting in a low signal magnitude. Thus, when choosing an inspection frequency ω, it is recommended that the flaw depth is contained within at least δ [14]. 

It is often the case that component flaws are in the vicinity of other artifacts resulting in overlapping eddy current signal response. For example, in steam generator tube inspection, tube degradations caused by surrounding ferromagnetic support structures will occur [29]. By principle of superposition, the resulting tube flaw and support structure will produce two overlapping signals. Multifrequency mixing is an ECT signal analysis technique that uses two or more frequencies simultaneously to filter out undesired artifacts from a base frequency signal $S_{Base}$. One way to implement multifrequency mixing is outlined in Avanindra [30] and Jung et al. [31]. The exact algorithm is not outlined here but a brief description is given. The assumption is that the $S_{Base}$ contains a linear superposition of flaw signal and artifact signal, while $S_{Aux}$ is less sensitive to the flaw and more sensitive to the artifact [30,31]. A linear transformation A applied to $S_{Aux}$, containing a rotation and a total scale, is used to match the artifact signal in $S_{Base}$. Specifically, an error minimization algorithm is applied to a loss function given by:

(6) $$L={\left|S_{Base}-AS_{Aux}\right|}^{2}.$$

Once a minimum is found, $S_{Aux}$ is subtracted from $S_{Base}$ leaving behind the eddy current response with the artifact removed, ideally representing the flaw indication.

### 1.3. Finite Element Modelling

All finite element method (FEM) modelling was done using COMSOL Multiphysics version 6.2 on a personal computer (PC). Specifications of the PC include an i5 12400f Intel Core processor (6 cores) and 16 gigabytes of DDR4 RAM. Given the ability to marginally detect a sub-millimeter near surface flaw with an ECT array probe, coil parameters were measured and used for FEM as starting parameters in Table 1.

Consideration was given to determine the appropriate coil size and probe design to increase SNR and thus detectability. The best compromise between resolution and signal amplitude is when the coil height and coil diameter are equal to the defect depth [32,33]. However, the array probe specified in Table 1. would be too small to give an adequate depth of penetration [14]. To maximize performance for a flaw located at a depth of 1 mm, the coil outer diameter and height were increased to 2 mm and simulated in FEM. A T/R configuration was used as it has increased SNR compared to conventional impedance eddy current probes, is directionally dependent, making it possible to resolve flaw orientations such as circumferential and axial cracks, and has a greater depth of penetration, which varies with the size of the coil and coil separation [17,20,25]. T/R coils are also less sensitive to changes in lift-off [20]. A ferrite core was also modelled, due to its increased effectiveness compared to an air core. Ferrite provides a higher flux density and hence a larger voltage response than an air core. Ferrite cores are known to reduce field spread, increase flux density [34] and improve SNR [35]. For this application, it was proposed that a 1 mm skin depth was appropriate as all target flaws would be within this depth. For a sample of Ti5553, the relative permeability and resistivity are *μ_r_* = 1.00005 [36], *ρ* =181 ± 1 µΩ·cm (as measured in this paper), respectively. Resistivity of the AM Ti5553 was measured using a four-point method summarized later in this study. Substituting *μ_r_*, *ρ* along with the 1 mm requirement into Equation (4). and solving for the frequency required to reach a minimum of 1 mm skin depth gives a test frequency of 448 kHz. Using Equation (5). and solving for $f_{90}$ yields 354 kHz, which was used for further modelling and optimization.

**Figure 1:**
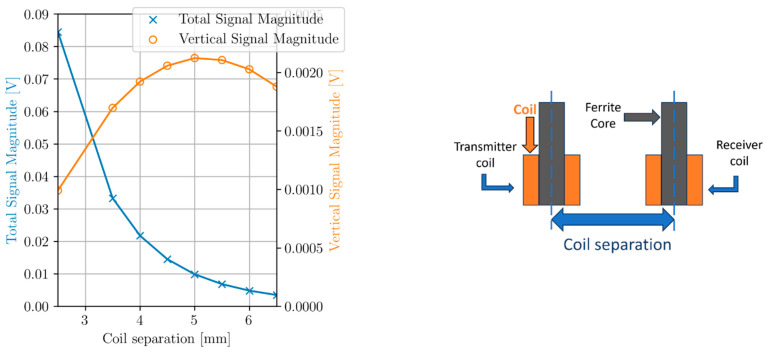
FEM comparison between maximum total signal magnitude and maximum vertical signal magnitude with increasing coil separation and constant test frequency of 354 kHz.

**Figure 2:**
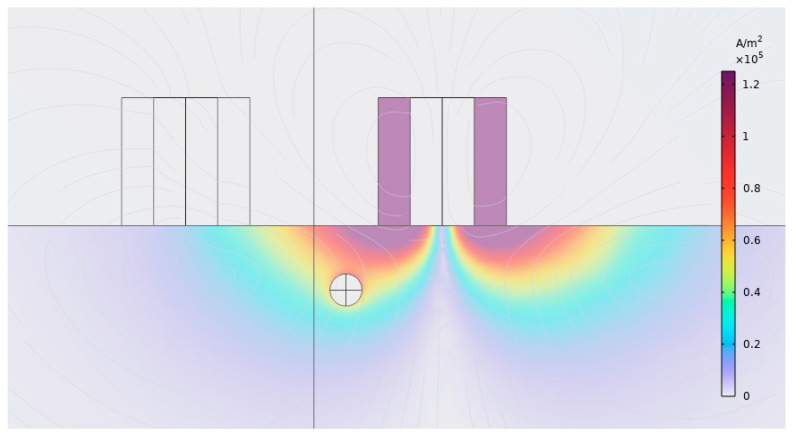
Eddy Current density flow around simulated spherical defect with optimal design parameters and 0.1 mm lift-off. This figure is a screen shot taken from COMSOL.

Figure 1 shows a FEM simulation of receive coil response due to a spherical void 500 µm in diameter and 1 mm below inspection surface. As coil separation increases, an exponential decrease in maximum total signal magnitude is observed, attributed to the change in coil impedance due to the presence of the void. However, the maximum vertical signal magnitude increases initially, peaks at 5 mm separation, and then decreases thereafter, with increasing coil separation. While a coil separation of 5 mm would be optimal to maximize SNR, the total signal magnitude becomes small at this separation. If a coil separation of 4 mm is considered, approximately twice the signal magnitude of a 5 mm separation is achieved with only a 9.4% loss in SNR. Thus a 4 mm coil separation was chosen. Figure 2 shows flow of EC density, *J*, around a simulated spherical void with selected design parameters, at 0.1 mm liftoff as simulated in COMSOL FEM. Some enhancement of EC density immediately above the void, compared with the no void case on the right, is observed. 

**Figure 3:**
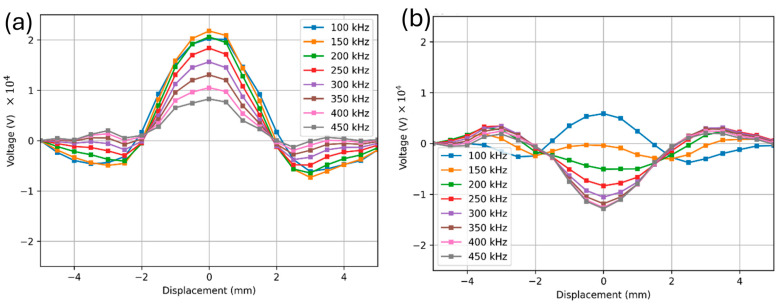
FEM simulated response at various frequencies due to a spherical void with 4 mm coil separation and 0.1 mm liftoff. (**a**) shows the vertical component of pickup coil voltage and (**b**) shows the horizontal component.

The FEM simulated frequency analysis was completed at constant coil separation of 4 mm at 0.1 mm lift-off to investigate impedance response of a 500 µm diameter spherical void 1 mm below the surface. Figure 3a shows vertical response to a spherical defect located at 0 mm displacement and Figure 3b shows the horizontal response, both at frequencies between 100 kHz and 450 kHz. The maximum vertical signal was calculated to be at 100–150 kHz. This was not in agreement with the frequencies calculated using Equation (5) [14] nor is it in agreement with some of the experimental results obtained in later sections. The type of flaw that is specifically described in [14] is a far side subsurface flaw, or a flaw that is exposed to the surface on the far-side of the inspection coil. In this case, eddy current cannot flow underneath the far side flaw. A void is fundamentally different from a far side or a near side flaw as current can pass above, around, or underneath of the flaw, as pointed out in the Introduction. Experimental data were compared to this observation later in this study to verify optimal frequency for vertical defect response. From FEM, a 100 kHz test frequency was chosen as an optimal frequency for inspecting 500 µm voids. The final probe dimensions and test frequencies are summarized in Table 1. 

## 2. Experimental Technique

### 2.1. Materials

A resistivity measurement of AM Ti5553 was performed using a standard four-point method. All measurements were taken at room temperature T=22.0 ± 0.1 °C. The mean resistivity was measured to be ρ=181 ±1 µΩ∙cm where the uncertainty was calculated for 1 standard deviation. Resistivity using an eddy current technique was used to verify the four-point method giving a value of $\rho_{ECT}=179\pm 3$ µΩ∙cm. These results are within 1% error of each other. 

The measured cross-sectional area was compared against a calculated cross-sectional area using the mass density value of AM Ti5553 from literature. From Ahmed et al. [37] the density of AM Ti5553 using Selective Laser Melting (SLM) was reported to be 4.67 g/cm^3^. The mass of the bar sample was 31.16 ± 0.01 g as measured using a digital mass scale. Using the measured value of $L_{total}$ and density from literature, the cross-sectional area was calculated to be 9.573 ±0.001 mm^2^ resulting in a 2.7% percent difference with respect to the measured A. The lower calculated cross-sectional area suggests the AM Ti5553 samples from this report have a lower density than that of Ahmed et al. [37], hence, DLD may result in lower density Ti5553 compared to SLM. Possible reasons for this lower density may be due to increased porosity when DLD is used. The possibility of increased porosity further motivates the need for NDT of Ti5553 components. 

**Figure 4:**
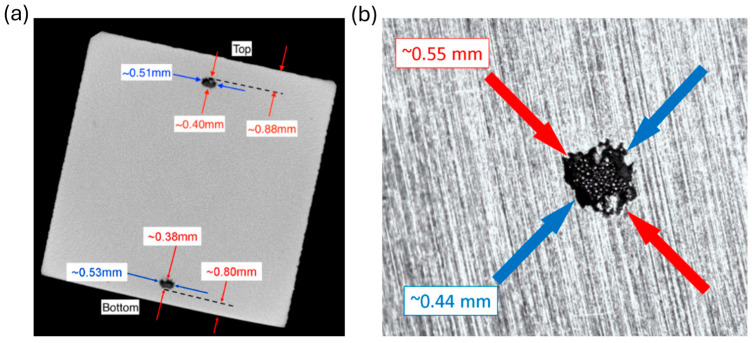
Images of physical flaws. Radiograph image in (**a**) shows the sub-surface cavities found in Samples C. Image (**b**) shows an exposed sub-surface void from Sample A.

**Figure 5:**
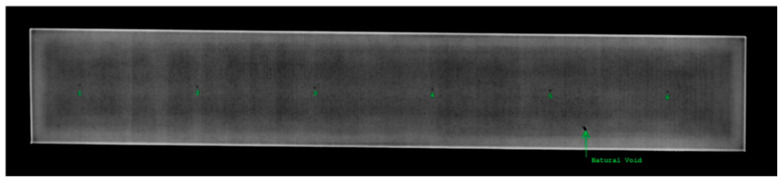
Radiograph of Sample A showing the locations of 6 sub-surface voids.

Sub-surface voids added into the Ti5553 Samples C and A during the AM process are shown using radiography in Figure 4a and an exposed void from Sample A in Figure 4b after milling. Figure 5 shows a radiograph of 6 the unexposed sub-surface voids in Sample A. Dimensions of all samples used, and their associated flaws are shown in Table 2.

**Figure 6:**
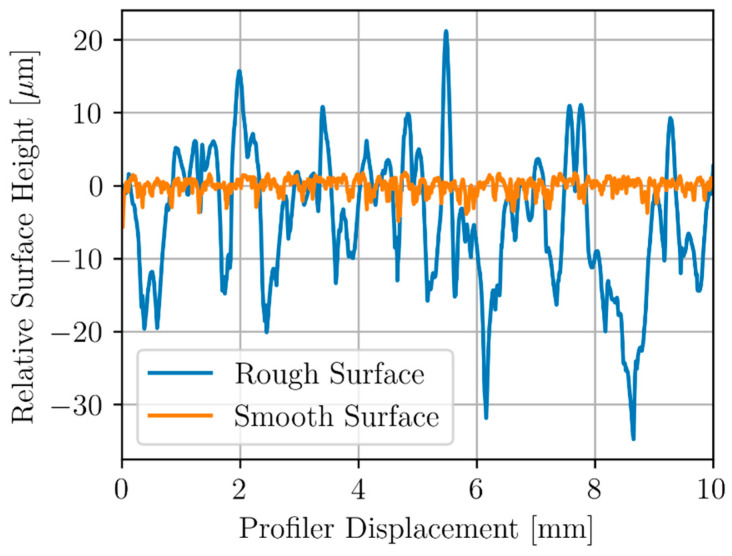
Relative surface height profile of sample c taken along both the smooth and rough inspection surfaces.

To characterize surface roughness, a Bruker Dektak XT (Billerica, MA, USA) surface profiler was used to measure the surface roughness of Sample C by taking a 10 mm linear scan of the surface topography. Figure 6 shows the variation in surface height for both the top and bottom surface of Sample C.

The rough surface of Sample C showed a variance of approximately 80 µm while the smooth side had a variance of 1 µm. Surface roughness of the inspection surface of Sample A was also measured and showed surface roughness very similar to that of the Sample C’s rough surface.

### 2.2. Probe

**Figure 7:**
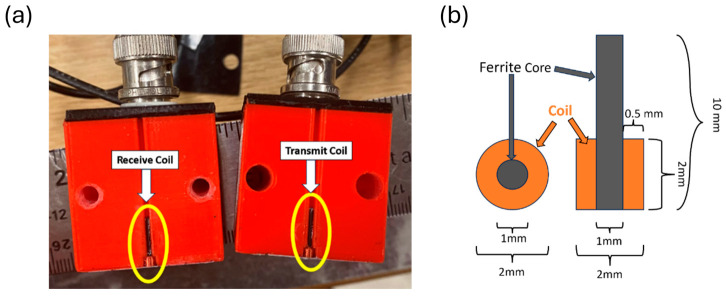
(**a**) Image of separated transmit receive probe (Cube Probe). The Cube Probe was 3D printed using PLA. The two halves can be bolted together to adjust transmit-receive coil separation and (**b**) a graphic showing the dimensions of a single coil from the probe.

Experiments demonstrated capability in detecting the nearest subsurface side-drilled hole in Sample B of Ti5553 with a 32 coil Olympus R/D Tech SBBR-025-01M-032 Eddy Current Array probe. This array could detect one subsurface side drilled hole with a 0.58 mm diameter at a depth of 0.75 mm below the inspection surface. All other defects, including 500 µm diameter subsurface voids, were not detectable with the array probe. The new probe built for this application was based on the FEM calculations, with dimensions listed in Table 1. An Adams-Maxwell Winding Systems coil winder was used to wind 300 turns of 44 AWG wire around a 1 mm drill bit. The coil was removed from the drill bit and a 1 mm ferrite core was placed through the coils’ inner diameter. The design of the probe consists of two 3×3×3 cm^3^ 3D printed polylactic acid (PLA) cubes containing one coil each. Plastic shim, approximately 0.10±0.01 mm in thickness, was secured to the bottom of the probe and the coils were set flush against it. The two halves can be screwed together; thin sheets of plastic can be inserted between them to vary transmit-receive coil spacing. Figure 7 shows an image of the two separated halves of the Cube Probe.

The resonant frequency of the two coils was measured using a Keysight oscilloscope and a function generator. It was essential to measure this value such that operating frequencies are not at resonance. To measure the resonant frequency of each coil, a coil was placed in series with a 2.7 kΩ resistor, then the frequency response of the series resistor was measured (100 kHz to 2 MHz in 100 kHz steps). The frequency that minimized the peak voltage drop across the resistor for each coil was approximately 1200±50 kHz, thus giving the resonant frequency. This process was done for each coil in the transmit-receive configuration. 

### 2.3. Apparatus

**Figure 8:**
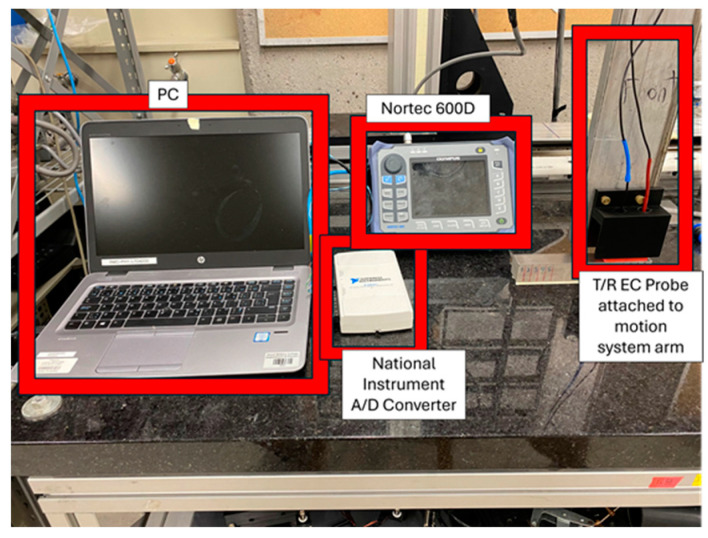
Image of experimental setup. The probe was translated using the motion system. Measurements were then collected from the Nortec 600D and stored on a PC via A/D converter.

Probe positionning was achieved using a THK Company Limited (Tokyo, Japan), 3-axis motion system to translate the probe across the samples. The motion system was controlled using the Galil Design Kit software version 2.0.7 where one can control displacement, speed, and acceleration. Translation occurred at a speed of 5 mm/s. The scanning speed was achieved by acclerating the arm from rest at a rate of 5 mm/s^2^. The probe was spring loaded such that the coils would remain in contact with the sample’s surface throughout translation. A SP1-50 linear string potentiometer was fastened to the motion system to accurately measure probe position during translation. An Olympus Nortec 600D was used to measure the change in voltage of the receive coil; all voltage measurements were passed to a National Instrument Digital I/O USB-6210 analog-to-digital converter and then displayed on LabVIEW for data acquisition and further analysis. Data was sampled at a rate of 1000 points per second. Line scans of the top surface were made to differentiate defect signal and noise signal (no defect signal). In this report, change in coil voltage due to lift-off was measured and then rotated by an arbitrary phase such that the lift off trajectory lay flush along the negative horizontal axis of the instrument screen, as per the standard ECT convention. Figure 8 shows an image of the experimental setup described above.

## 3. Results and Discussion

### 3.1. Sample D

**Figure 9:**
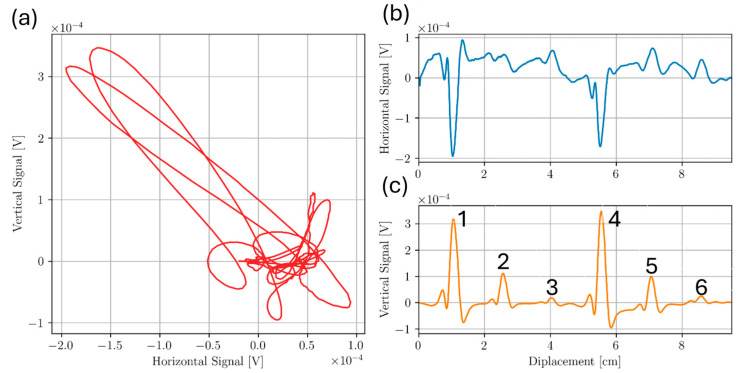
Scan of Bottom drilled holes Sample D at 350 kHz where (**a**) shows the Lissajous plot and (**b**,**c**) show horizontal and vertical components of (**a**), respectively. Hole numbers 1–6 are next to vertical response component in (**c**) indicating the location of bottom drilled holes.

**Figure 10:**
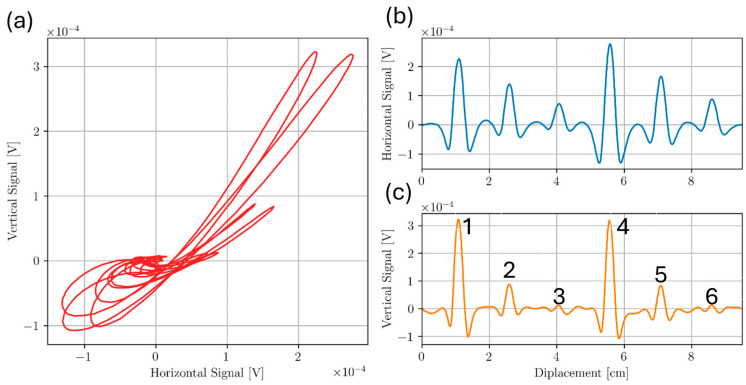
Scan of Bottom drilled holes Sample D at 100 kHz where (**a**) shows the Lissajous plot and (**b**,**c**) show horizontal and vertical components of (**a**), respectively. Hole numbers are next to vertical response component in (**c**) indicating the location of bottom drilled holes.

For the Ti5553 plate, line scans were taken across the top surface of the sample directly above all defects. Transmit receive scanning configuration was set so that the receive coil would follow the drive coil as the probe was scanned along length of sample. This configuration was chosen as it gave the largest response to defect signal as an approach signal was observed using this configuration, resulting in a larger signal overall. Frequencies from 100 kHz to 450 kHz were chosen in 50 kHz increments to determine the optimal frequency for detecting each defect. For each frequency, liftoff signal was rotated to lie along the negative horizontal axis. Approximate locations of Holes 1, 2, 3, 4, 5, and 6 were 1.0 cm, 2.5 cm, 4.0 cm, 5.5 cm, 7.0 cm and 8.5 cm along the probe displacement scale, respectively. For each graph displayed in this report, line scans were repeated 3 times at a sampling rate of 1000 datapoints per second. The data was binned in bins of 0.5 mm interval over the entire scan length. Each data point graphed is the average over 3 scans after initial binning in each trial. The uncertainty is the standard deviation for these averages. Figure 9 and Figure 10 show measured signals from the 350 kHz and 100 kHz test frequencies, respectively. The results show clear indications of the flaws at the specified defect locations. Defect signal phase was analyzed from the lissajous plots for the 350 kHz case. For Holes 2 and 4, phase seperation θ between the lift-off signal response and the max defect amplitude signal (angle formed from the negative horizontal axis to the null point and the peak in the flaw signal amplitube to the null point), was measured. Qualitatively, using $\theta =2\beta =2\frac{z}{\delta }$, flaw depth z can be approximated [14]. The estimated flaw depths of Holes 2 and 4 were 1.16 mm and 1.25 mm, respectively, with percent errors from the true depths of 20% and 30%, respectively. Additionally, the signal response for Holes 2 and 4 shows to be mostly vertical, indicating good flaw discrimination. 

From the results in Figure 9 and Figure 10, a decreasing signal amplitude is observed with increased flaw depth from the inspection surface, as expected. The results suggest the T/R ECT probe can detect sub-millimeter drill holes below and near the inspection surface. However, at higher frequencies such as 350 kHz, more noise is observed in the horizontal component of the signal, suggesting the EC probe’s sensitivity to surface roughness has a significant liftoff component. The surface roughness of Sample D had a surface roughness variance of 1 µm, like the smooth surface of Sample C, and the results show responses with minimal noise. The effects of increased surface roughness on ECT signal response will be investigated further later in this paper. 

### 3.2. Sample A

**Figure 11:**
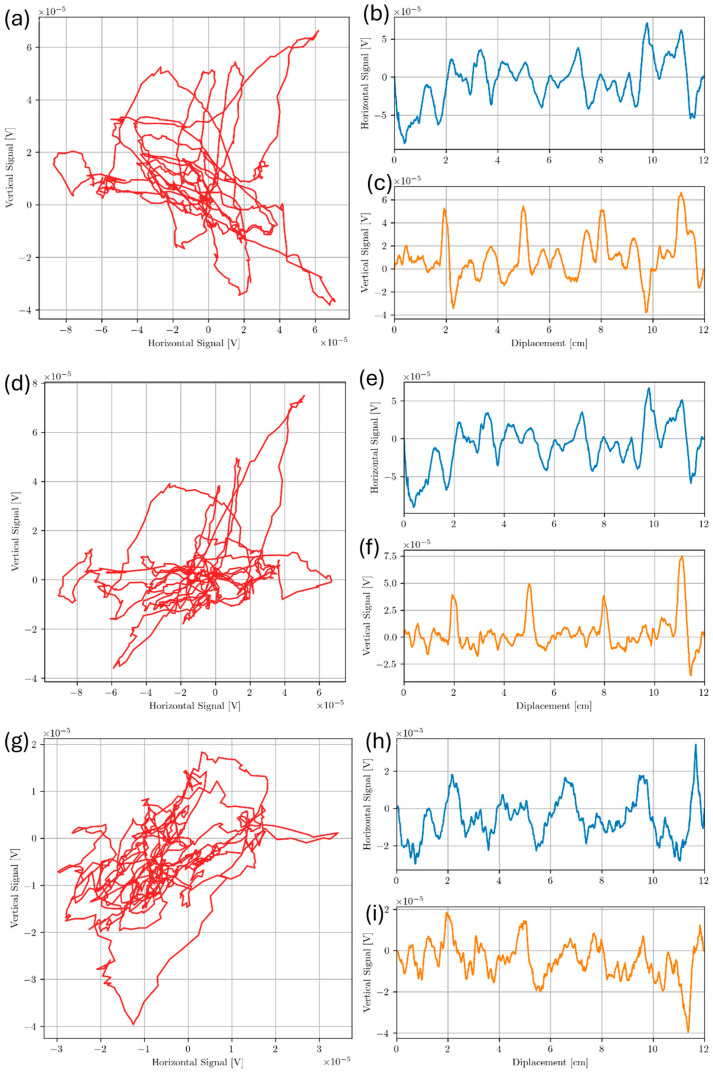
Scan of sub-surface voids from Sample A. Lissajous plot, horizontal and vertical components are shown for each of 300 kHz in (**a**–**c**), 350 kHz in (**d**–**f**), and 400 kHz in (**g**–**i**), respectively.

As with the bottom drilled holes, frequencies from 100 kHz to 450 kHz in 50 kHz increments were chosen to scan Sample A. It was found that most frequencies in this range gave no indication of any defect being present. Approximate locations of Voids 2, 3, 4 and 5 are at 2 cm, 5 cm, 8 cm and 11 cm along the probe displacement scale respectively. Only the inner 4 voids were measured (Voids 2 through 5) as scanning Voids 1 and 6 tended to increase probe tilt due to relatively large coil housing. Probe tilt is like lift-off but instead of coils being uniformly raised above a conducting surface, one side of the coil may be closer to the surface than the others. The effect of tilt is a change in impedance that may not be in the same direction as that of uniform lift-off [38,39]. If lift-off is in the horizontal direction, then a tilt may have a slight vertical component. Figure 11 shows line scans taken along the midline of Sample A at a test frequency of 350 kHz, which was the only frequency to show clear indication of flaws separated from lift-off. 

At a test frequency of 350 kHz, the vertical component of the signal shows 4 distinct peaks at the approximate void locations. Below at 300 kHz and above at 400 kHz do not show these same peaks in voltage. For Sample A, signals measured at 300, 350, and 400 kHz (about the $f_{90}=$ 354 kHz) suggest that the $f_{90}$ in Equation (5) proposed in Cecco et al. [14] is a reasonable technique for distinguishing defect signal from lift-off variation. A possible reason for the high noise level in the signal is due to high surface roughness on the inspection surface. The effect of surface roughness was investigated further in the following section of this study.

### 3.3. Sample C

**Figure 12:**
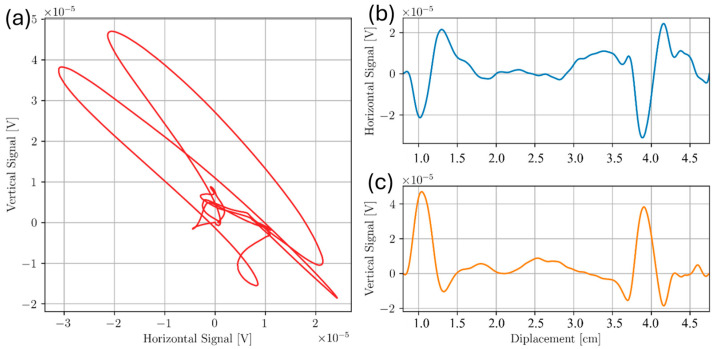
Scan of two subsurface voids below smooth inspection surface of Sample C at 300 kHz. A Lissajous plot shown in (**a**) was broken into both the horizontal (**b**) and vertical (**c**) component.

The results in this section show the effect of surface roughness on ECT. Figure 12 shows a line scan taken of Sample C at 300 kHz on the smooth inspection surface. Approximate locations of defects are at 1.0 cm and 4.0 cm along the probe displacement scale, respectively.

**Figure 13:**
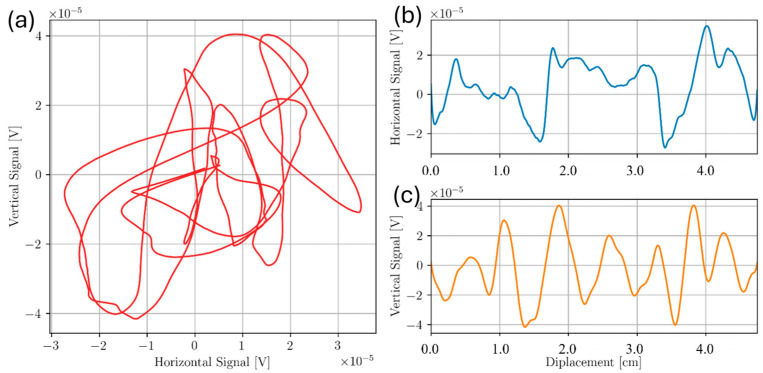
Scan of two subsurface voids below rough inspection surface of Sample C at 100 kHz. A Lissajous plot shown in (**a**) was broken into both the horizontal (**b**) and vertical (**c**) component.

**Figure 14:**
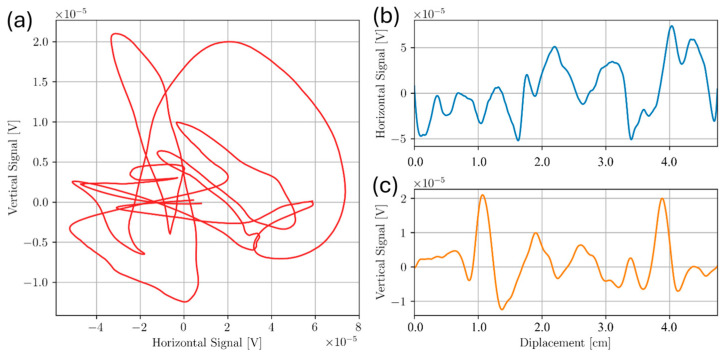
Scan of two subsurface voids taken below rough inspection surface of Sample C at 300 kHz. A Lissajous plot shown in (**a**) was broken into both the horizontal (**b**) and vertical (**c**) component.

Two distinct peaks in the vertical and horizontal component for 300 kHz on the smooth inspection surface were observed. These peaks lie within uncertainty of the recorded defect locations, indicating successful defect detection. In contrast, an identical experiment was conducted on the rough inspection surface. These results are shown in Figure 13 and Figure 14 for 100 kHz and 300 kHz, respectively. 

Line scans of the top and bottom surface of Sample C reveal the effect of surface roughness on ECT. When surface roughness is increased, greater variation in both the horizontal and vertical components was observed. For the rough surface at f= 100 kHz, the vertical component of the signal is mostly overwhelmed by noise despite liftoff having been rotated to the horizontal component. This would suggest surface roughness produces signal responses in addition to a simple lift-off component. Likewise, the vertical component at 300 kHz for the rough surface shows a similar signal response. For the smooth surface, the defect signal is pronounced in both the horizontal and vertical component at a lower frequency of 100 kHz. At 300 kHz, the defects are still represented by two distinct peaks in the vertical component, but not in the horizontal component. This may be due to the probe’s higher sensitivity to surface variation at the higher frequency based on the skin depth equation. 

**Figure 15:**
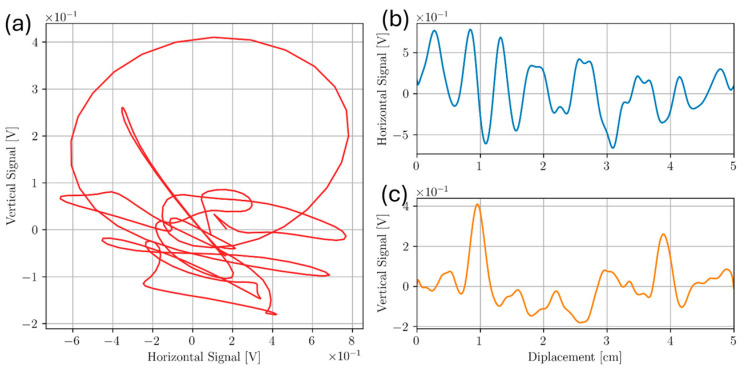
Results from a 300–750 kHz frequency mix from the rough inspection surface of Sample C. A Lissajous plot shown in (**a**) was broken into both the horizontal (**b**) and vertical (**c**) component.

Multifrequency mixing was done to filter out artifacts from the rough inspection surface. Since the skin depth is much smaller at higher frequencies, eddy currents are more concentrated towards the inspection surface. Thus, a greater sensitivity to surface roughness is seen at higher test frequencies. An auxiliary frequency of 750 kHz was chosen as it provides a small skin depth, while keeping far from the resonant frequency of the probe. Measurements were taken at 300 kHz and 750 kHz, simultaneously using the Nortec 600D. Results of the frequency mix are shown in Figure 15 using the method described earlier in this paper. 

The results from the frequency mixing show an increase in SNR from 4.3 to 6.5 for the subsurface void at 1 cm probe displacement. A slight decrease in SNR was measured for the flaw at 4 cm displacement from 4.6 to 4.3, however an average increase of 22% in SNR was seen for all flaws after mixing. 

Some potential sources of uncertainty arise, since the sub surface defects are only approximately spherical. From Figure 4, there is some irregularity to the edge of the exposed void that was not simulated in FEM. Additionally, the CT scan in Figure 4a reveals the depth of the voids but only for one pair of top and bottom voids in Sample C. The exact depths below the surface of the other voids are unknown. A new experimental setup should be designed to improve constant probe contact with the sample surface. The relatively large probe housing may be preventing the coils from maintaining constant liftoff from the scanning surface, due to potential nonplanar variations arising during the printing process. Effectively, the relatively large probe housing resulted in increased tilt and liftoff due to sample warp. As stated earlier in this paper, only 80% of the subsurface voids were detected due to the increased probe tilt while scanning voids close to the ends of Sample A. It is proposed that a transmit receive probe with a smaller base be built and tested. Some other challenges in ECT involve accurately detecting depth of subsurface voids. Since all voids were printed at nominally equivalent depths, no calibration curve could be made to infer flaw depth. As future work, a series of sub-surface voids should be printed at various depths close to the inspection surface for creating a flaw depth calibration curve. Lastly, as mentioned in the Introduction, porosity on the order of 500 µm is considered large for modern AM techniques. During the AM process, flaws could be introduced closer to the surface before being buried by a subsequent layer. If a T/R probe optimized for flaw detection were used in-situ to monitor for porosity, these types of flaws would result in a larger EC signal response in accordance with the skin depth, Equation (4). In this case, it is conceivable that smaller defects on the order of 100 µm could be detected with a T/R probe at sufficient SNR.

## 4. Conclusions

It was demonstrated in this report that 1 mm deep 500 µm diameter voids can be detected in additively manufactured direct laser fabricated (DLF) Ti5553 using transmit-receive eddy current inspection. These voids present an inherent problem for DLF and successful detection of these defects can aid in non-destructive component inspection. The transmit-receive probe from this study could detect 80% of the AM voids subject to surface roughness and sample warpage. Direct comparison between measured signal and FEM was challenging as modelling irregularly shaped voids and random surface roughness was not feasible. A surface roughness variance of 80 µm on the Ti5553 samples presented a considerable challenge when comparing experimental results to those predicted by FEM. Random fluctuations in the eddy current signal response arose due to surface roughness, especially at higher test frequencies where skin depth was reduced, and coil impedance was more affected by near-surface effects. Based on results measured for 500 µm sub-surface voids 1 mm below a relatively smooth surface, it is conceivable that smaller diameter voids can be detected with this probe. Multifrequency mixing attempting to remove the effects of surface roughness improved SNR by 20% in this study. 

## Figures and Tables

**Table 1:** Coil parameters for all FEM modelled coils and physical coils used in this report.

Coil Parameters	ET Array Probe	FEM Starting	FEM Optimized	Physical Probe
Coil OD (mm)	1.5 ± 0.1	2	2	2.50 ± 0.03
Coil ID (mm)	0.5 ± 0.1	1	1	1.00 ± 0.03
Coil Height (mm)	1.4 ± 0.1	2	2	2.0 ± 0.1
Wire Gauge (AWG)	44	44	44	44
Number of turns	320	300	300	300
Frequency (kHz)	1000	354	100	Various
Coil-Coil separation (mm)	2.0 ± 0.1	2.5	4	4 ± 0.1

**Table 2:** Sample dimensions with number, type, and dimensions of the associated flaws. For the side drill holes in Sample B, depths are measured from the inspection surface to the center of the drill hole. For the bottom drill holes in Sample D, depths represent the distance from inspection surface to the tip of the drill hole.

Ti5553 Sample	Dimensions (Height × Width × Length)	Flaw Number and Type	Flaw Dimensions		
A	3 × 3 × 19 cm^3^	12 subsurface voids	500 µm nominal diameter		
B	3 × 3 × 19 cm^3^	10 side drilled holes	Side Drill Hole Number	Diameter (mm)	Depth from inspection surface (mm)
			1	0.58	0.75
			2	0.58	1.25
			3	0.58	1.75
			4	0.58	2.25
			5	0.58	2.75
			6	0.79	3.25
			7	0.79	2.75
			8	0.79	2.25
			9	0.79	1.75
			10	0.79	1.25
C	1 × 1 × 7 cm^3^	4 subsurface voids	500 µm nominal diameter		
D	3 mm × 3 cm × 19 cm	6 bottom drilled holes	Bottom Drill Hole Number	Diameter (±0.008 mm)	Depth from inspection surface (±0.02 mm)
			1	0.787	0.46
			2	0.787	0.96
			3	0.787	1.46
			4	0.991	0.46
			5	0.991	0.96
			6	0.991	1.46

## Data Availability

Data is contained within the article.
